# Resectable fusiform internal jugular vein aneurysm with vascular excision and bypass with an 8 mm Maquet graft: A case report

**DOI:** 10.1097/MD.0000000000033582

**Published:** 2023-04-21

**Authors:** Ting-Sheng Gong, Tzong-Shiun Li

**Affiliations:** a College of Medicine, Chung Shan Medical University, Taichung, Taiwan; b Division of Cardiovascular Surgery, Department of Surgery, Changhua Christian Hospital, Changhua County, Changhua, Taiwan; c College of Medicine, Fu Jen Catholic University, New Taipei City, Taiwan.

**Keywords:** artificial graft, bypass, excision, internal jugular vein, venous aneurysm

## Abstract

Venous aneurysm is a rare vascular disease. Studies reveal that patients with fusiform internal jugular vein aneurysms are not suitable for embolization.

**Patient concerns:**

Two treatment options are considered, either bypass the aneurysm via stenting or excision of the lesion site and anastomosis using an artificial graft. The advantages of excision bypass include the absence of endoleak and stent migration; however, a larger wound and longer operation time are required for it.

**Diagnoses:**

Physical examination revealed a swelling in the right neck area. Head and neck computed tomography was performed for the survey. It revealed a 27.22 × 25.07 × 58.17 mm internal jugular fusiform aneurysm.

**Interventions:**

Vein excision was performed while using hemoclamps to control inflow and outflow, and a graft was anastomosed for bypass using an 8 mm Maquet graft.

**Outcomes:**

The wound recovery was favorable, with no signs of infection, and the pain was under control.

**Lessons:**

The patient had a contrast-enhanced head and neck computed tomography, and the images efficiently diagnosed a venous aneurysm. This patient had refractory pain, which was a significant indication of the operation. We decided by ourselves on the duration of the interval of following up. We used excision and bypassing with graft, and the result was beneficial.

## 1. Introduction

Venous aneurysm is a rare vascular disease, and based on literature it was first documented in 1928 by Harris RI.^[1]^ The most common sites of venous aneurysms are the upper extremities and they are usually asymptomatic.^[2]^ Laryngocele, branchial cyst, upper mediastinum tumor or cyst, cystic hygroma, thyroglossal cysts, dermoid cyst, cervical adenitis, and metastatic adenopathy are often suspected when patients present with a neck mass swelling.^[3]^ The secondary causes of venous aneurysm include previous trauma, inflammation, degenerative vessel wall changes, and increased vascular pressure, including that in varicose veins.^[4,5]^ Literature states that patients with fusiform internal jugular vein aneurysms are not ideal for embolization. Therefore, 2 treatment options are considered here, either bypass the aneurysm by stenting or excise the lesion site and anastomosis using an artificial graft. This was a relatively rare case; thus, we documented its outcome and management for future reference and studies.

## 2. Patient information

A 47-year-old woman presented with a history of arrhythmia without regular medication control. On May 5, 2020, she was admitted to the emergency department due to swelling and a painful neck mass on the right side, which started 1 week ago. She previously underwent conservative treatment at a clinic, but the symptoms did not subside. Moreover, she had refractory pain with painkiller use.

## 3. Clinical findings

Physical examination revealed a swelling in the right neck area. she stated that her neck pain was over the right mandible, and it was radiating to the head and pterion area. The symptoms worsened when swallowing. She was hemodynamically stable and her vital signs included a temperature of 36.1°C, pulse rate of 105 beats/min, respiratory rate of 20 breaths per minute, blood pressure of 138/114 mm Hg, Glasgow Coma Scale of E4M6V5, and visual analog scale of 6/10.

## 4. Timeline

### 4.1. Diagnostic assessment

**Figure 1.**
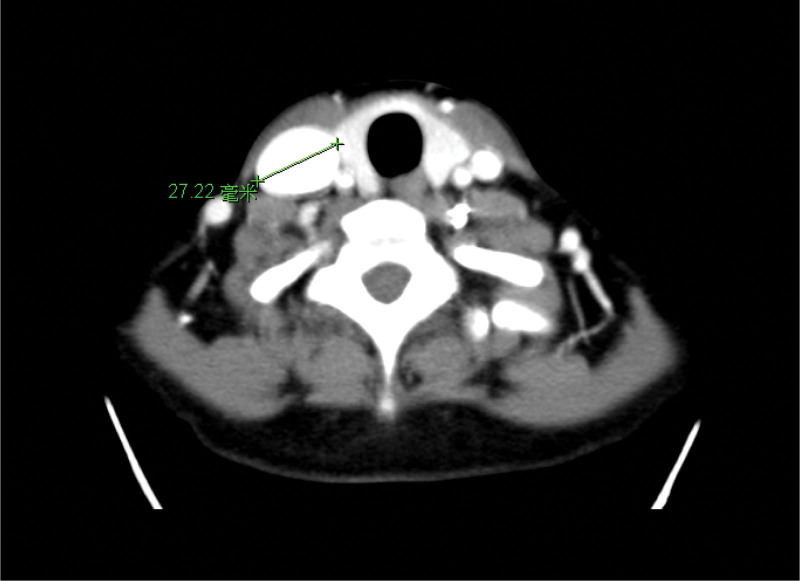
Axial view of head and neck CT showed a right fusiform internal jugular vein aneurysm, 27.22 mm in maximum diameter. CT = computed tomography.

**Figure 2.**
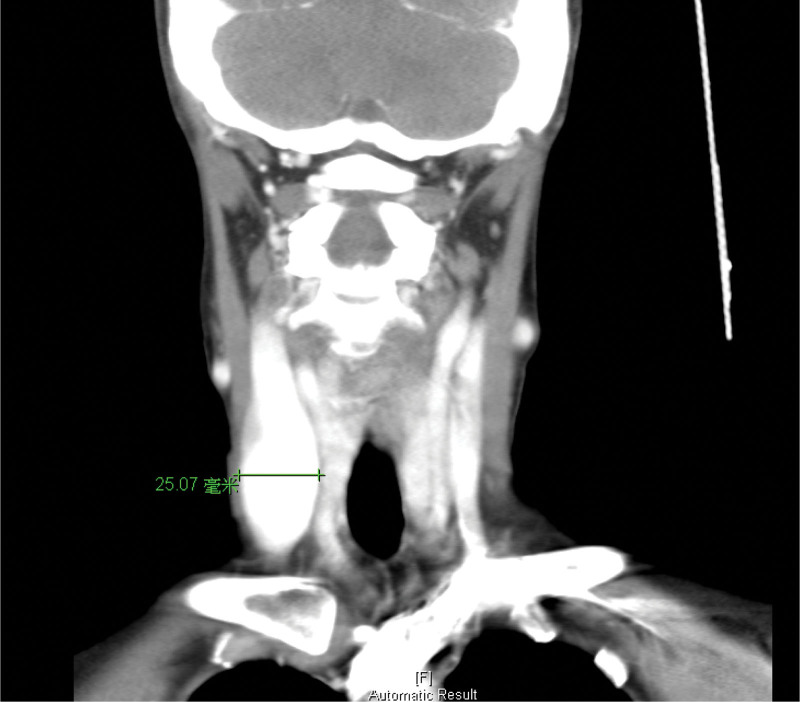
Coronal view of head and neck CT showed a right fusiform internal jugular vein aneurysm, 25.07 mm in maximum width. There was a filling defect, which was a thrombus. CT = computed tomography.

**Figure 3.**
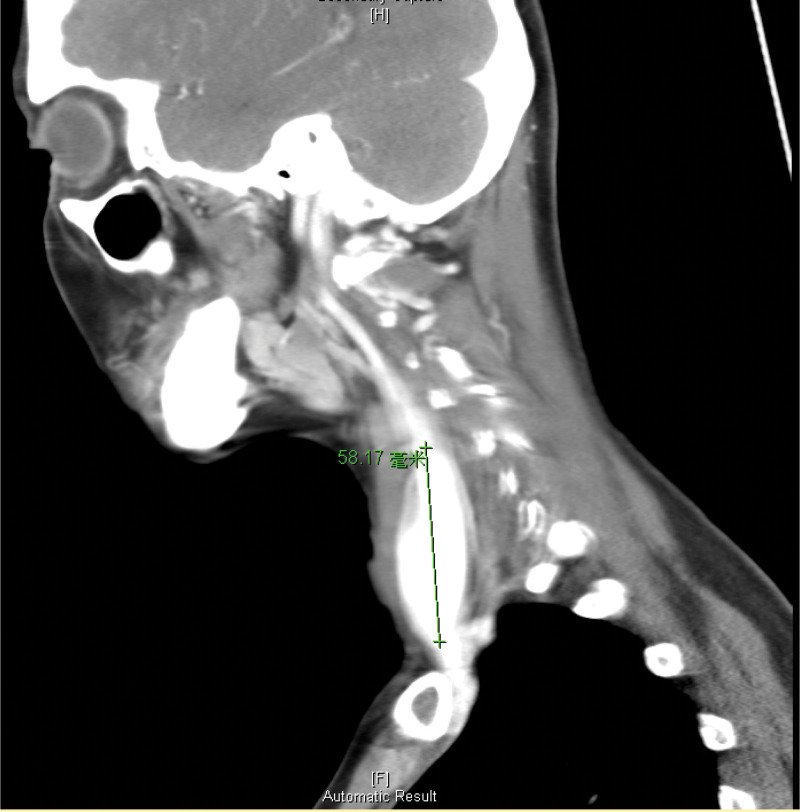
Sagittal view of head and neck CT showed a right fusiform internal jugular vein aneurysm, 58.17 mm in maximum height. CT = computed tomography.

Laboratory tests revealed no leukocytosis (white blood cells: 7400 10^3^/µL, neutrophils: 42.5 %) and mildly elevated glutamate pyruvate transaminase (60 U/L). Head and neck computed tomography (CT) was performed for survey and to exclude deep neck infection. It revealed a 27.22 × 25.07 × 58.17 mm internal jugular fusiform aneurysm (Figures 1–3). The right nodule measuring 9.1 mm showed normal fatty and solid content similar to a reactive lymph node.

### 4.2. Therapeutic intervention

**Figure 4.**
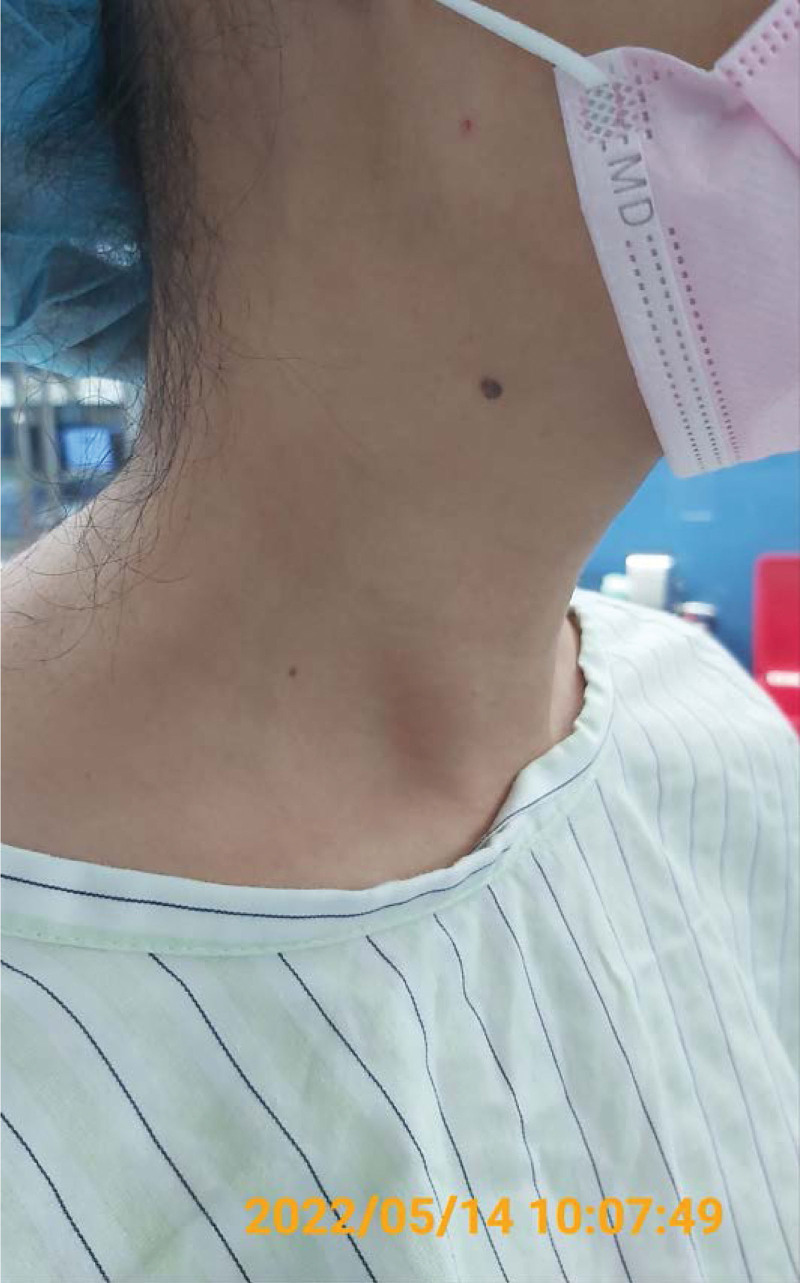
Appearance of the right neck mass before the operation. The patient was in a standing position.

**Figure 5.**
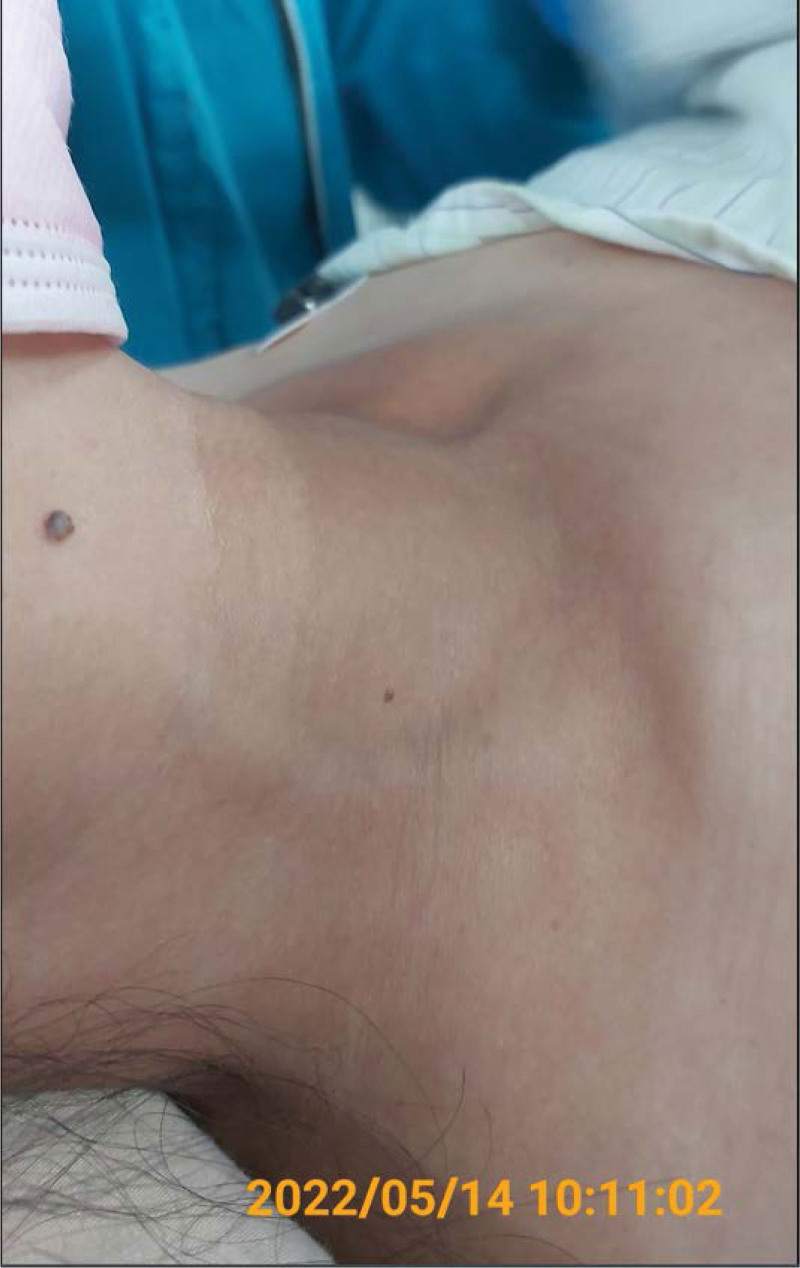
Appearance of the right neck mass before the operation. The patient was in a supine position.

**Figure 6.**
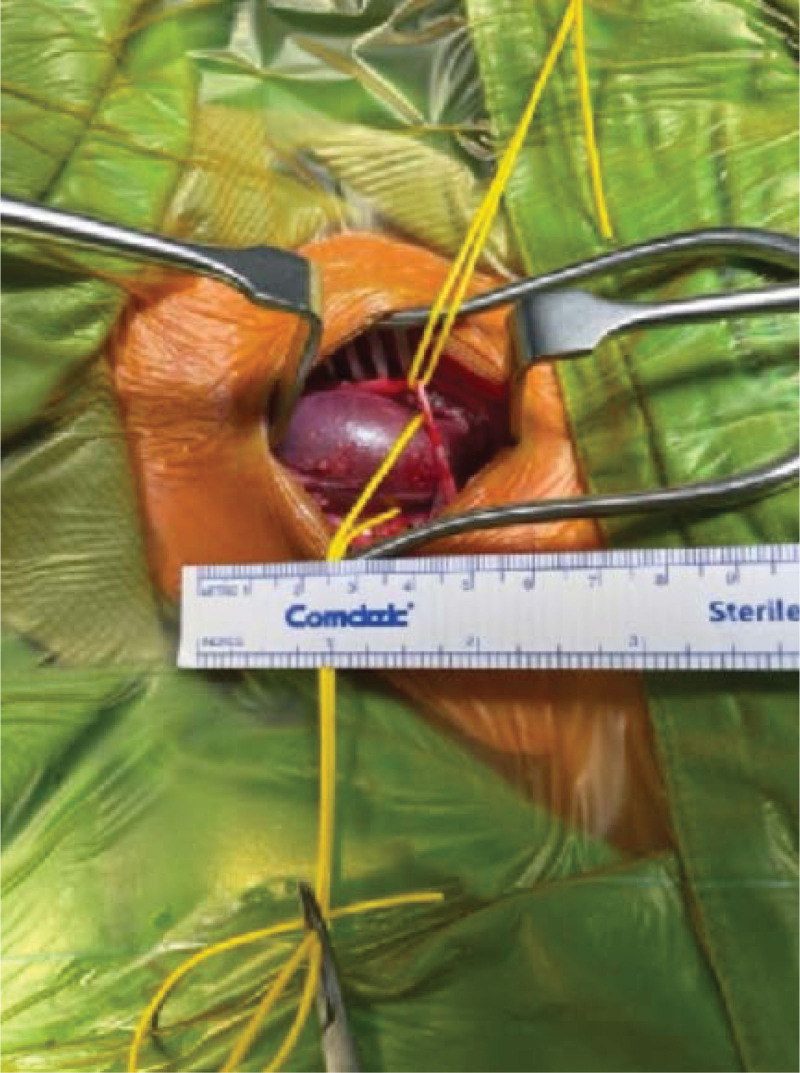
Well-defined dissection of the aneurysm.

**Figure 7.**
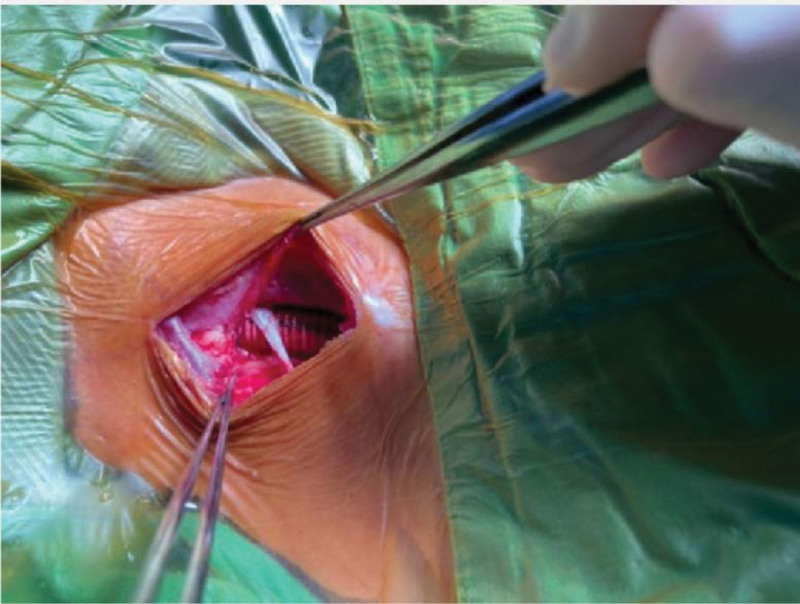
There was the post-excision and graft anastomosis.

We were consulted and the management protocol was explained to the family. This included placing a stent to bypass the fusiform aneurysm or excising the lesion site and grafting a substitute for the original vein. Subsequently, medications for pain control were prescribed and she was instructed to follow up at the outpatient department (OPD) for scheduled surgery. On May 6, 2022, her condition was evaluated and she chose to excise the lesion via artificial graft. On May 14, 2022, the patient was admitted to the operating room under general anesthesia (Figs. 4 and 5). A peri-sternocleidomastoid skin incision was conducted, and a well-defined dissection of the aneurysm was performed. Vein excision was performed while using hemoclamps to control inflow and outflow, and a graft was anastomosed for bypass using an 8 mm Maquet graft (Figs. 6 and 7). After hemostasis, the wound was closed layer by layer. The total time required for the surgery was 98 minutes and the estimated blood loss was 3 mL. Subsequently, she was admitted to the ward for further care.

### 4.3. Follow-up and outcomes

After the operation, oral cephalexin (500 mg) every 12 hours for 3 days was prescribed to prevent infections, while acetaminophen and nalbuphine were prescribed for pain control as required. The wound dressing was changed daily. The wound recovery was favorable, with no signs of infection, and the pain was under control. The patient was discharged 4 days after admission. The pathological report was right internal jugular vein resection of the aneurysm. The specimen submitted consisted of 1 fresh tissue fragment measuring 3.4 cm in length and 1 cm in diameter. Grossly, it was dilated and tortuous. Microscopically, the section was a tortuous vein with dilated lumen, consistent with the features of aneurysm. On June 1, 2022, she followed up at our OPD, and the wound was dry and clean on observation.

## 5. Discussion

Diagnostic tools for aneurysm include duplex ultrasonography scanning, 3-dimensional ultrasonography, CT, magnetic resonance imaging with magnetic resonance angiography/magnetic resonance venography, and catheter-directed venography.^[6,7]^ In our case, the patient had a contrast-enhanced head and neck CT, and the images efficiently diagnosed a venous aneurysm.

Surgery was considered due to cosmetic reasons, thrombus formation, pulmonary embolism, spontaneous rupture, and thrombophlebitis.^[8]^ This patient had refractory pain, which was a significant indication of the operation. Regarding venous aneurysm, surgical methods included either ligation of the aneurysm if the lesion is saccular or bypassing the lesion with artificial graft if the venous aneurysm can be excised. We considered the size and site of the lesion to confirm its patency and prevent brain edema.^[9]^ The 2 methods were explained to the patient. The first method was excision and bypass with graft, and its advantages include the absence of endoleak and stent migration problems, but it would require a larger wound healing and operation time. The second method was using a stent bypass, with opposite pros and cons. Moreover, the patient needs to receive a radiation dose and contrast use when using the endovascular method. We also need to observe the position of the stent to verify migration.

Two weeks later, the patient was present at our OPD for follow-up. There are no significant studies on the long-term outcomes of patients who received different treatments for internal jugular vein aneurysms.^[10]^ We decided by ourselves on the duration of the interval of following up. We monitored the visual analog scale score, appearance, vessel patency, and pain control medication to evaluate the recovery rate.

The lesion was found incidentally when undergoing a CT scan. She denied a history of trauma; moreover, infection, hereditary familial diseases, and autoimmune problems were ruled out. The study presented a focal thickening of the intimal layer, an increase in the amount of connective tissue, and an increase in endothelial cells compared to veins with non-aneurysmal components in the microscopic field.^[11]^ The pathological report of this patient also confirmed that the lesion was a simple venous aneurysm over the right jugular vein.

In conclusion, we used excision and bypassing with graft, and the result was beneficial. Furthermore, no complications occurred. The patient was satisfied with the outcome. This experience can offer a considerable way to treat fusiform internal jugular venous aneurysm.

## Acknowledgments

We appreciated the patient allowed us to share her experience and whole record of management this time.

## Author contributions

**Resources:** Tzong-Shiun Li.

**Supervision:** Tzong-Shiun Li.

**Writing – original draft:** Ting-Sheng Gong.

**Writing – review & editing:** Ting-Sheng Gong.
